# Intra-articular delivery of geraniol encapsulated by pH/redox-responsive nanogel ameliorates osteoarthritis by regulating oxidative stress and inflammation

**DOI:** 10.1007/s10735-023-10163-4

**Published:** 2023-10-17

**Authors:** Jun Pan, Youzhi Cai, Chi Zhang, Sanzhong Xu

**Affiliations:** Department of Orthopedics, The First Affiliated Hospital, Zhejiang University School of Medicine, Hangzhou, 310003 China

**Keywords:** Geraniol, PH/redox-responsive nanogel, Osteoarthritis, Oxidative stress, Inflammation

## Abstract

Osteoarthritis (OA) remains a challenging condition due to limited drug bioavailability within the avascular and dense cartilage matrix. This study introduces a pH/redox-responsive nanogel for enhanced delivery of geraniol in OA therapy. We investigated geraniol's role in preventing chondrocyte matrix degradation and designed a pH/redox-responsive nanogel as a delivery platform. Our methods included Western blot, histological staining, and immunohistochemistry. Geraniol treatment reduced Keap1 expression while elevating Nrf2 and HO-1 levels, effectively inhibiting cartilage matrix degradation. The pH/redox-responsive nanogel further enhanced geraniol’s therapeutic impact. Our study demonstrates that geraniol encapsulated within a pH/redox-responsive nanogel mitigates OA by regulating oxidative stress and inflammation. This innovative approach holds potential as an effective OA therapeutic strategy.

## Introduction

Osteoarthritis (OA) is a common orthopedic disease, affecting a significant proportion of middle-aged and elderly individuals. Statistics indicate that 12% of this population experience varying degrees of OA (Pereira et al. 2015). In the United States, over 27 million people suffer from OA, with a prevalence of over 50% in individuals aged 60 and above (Martel-Pelletier et al. 2016). A study conducted by the US Centers for Disease Control and Prevention and the Harvard Department of Public Health identified OA as one of the five primary causes of disability in older adults (Mandl 2019). Due to its high incidence and chronic nature, OA imposes a substantial life burden on patients and creates a significant economic burden on families and society (Thomas et al. 2017).

OA is a whole joint disease that involves all joint tissues, including cartilage, synovial membrane, infrapatellar fat pad, meniscus, and subchondral bone (Scanzello and Goldring 2012). The progression of OA is thought to involve an imbalance between the anabolic and catabolic processes in the joint, leading to a breakdown of the extracellular matrix and an increase in pro-inflammatory cytokines (Zheng et al. 2021). Despite numerous recent studies on OA, current clinical strategies for treating the disease remain limited due to its complex pathological mechanism (Palazzo et al. 2016). In addition to physical rehabilitation, pharmacological interventions are limited to symptom relief, and joint replacement is the only treatment option available for improving joint function (Peat and Thomas 2021). Therefore, it is necessary to continue exploring the molecular mechanisms underlying OA pathogenesis and develop new, specific therapeutic targets and strategies.

Oxidative stress (OS) results from the negative effects of free radicals in the body and plays a crucial role in promoting and developing OA (Ansari et al. 2020; Zahan et al. 2020). Redox system imbalances can cause intra-articular pathology and inflammation. Reactive oxygen species accumulation can interfere with chondrocyte anabolism processes, promoting chondrocyte dysfunction and degeneration, disrupting chondrocyte metabolism, disrupting the balance of cartilage matrix production and degradation, and accelerating articular cartilage degeneration (Tang et al. 2017; Wang et al. 2020). As OA progresses, cartilage’s self-healing ability diminishes due to insufficient blood supply and innervation (Guilak et al. 2018). Chondrocytes’ functional activity is also continuously impaired, causing a decrease in their ability to secrete matrix components such as Col2a1 and aggrecan (Huang and Wu 2008).

Numerous studies have investigated the use of natural compounds to treat OA, including various terpenoids. For example, oleanolic acid, a triterpenoid found in many plants, has been shown to have anti-inflammatory and anti-oxidative effects on chondrocytes (Kang et al. 2017). β-caryophyllene, a sesquiterpene found in many essential oils, has also been reported to have anti-inflammatory and chondroprotective effects on OA (Mlost et al. 2022). In addition, ginkgolides, terpenoids extracted from Ginkgo biloba, have been shown to inhibit cartilage degradation and reduce inflammation in OA (Ma et al. 2022). Geraniol is a natural acyclic isoprenoid monoterpene that occurs in several plant essential oils, including rose, lemongrass, lavender, and lime (Maczka et al. 2020). It is classified by the U.S. Food and Drug Administration as “Generally Recognized as Safe” and has been approved as a food additive to adjust the flavor of beverages, candy, and ice cream (Cho et al. 2016). Recent research has shown that geraniol has diverse pharmacological properties, such as antioxidant, anti-inflammatory, anti-tumor, antimicrobial, liver protection, and neuroprotection (Lin et al. 2021a, b; Pavan et al. 2018; Zhang et al. 2019). Moreover, some studies have indicated that geraniol can regulate oxidative stress levels through the Keap1-Nrf2 pathway (Younis et al. 2020; Younis et al. 2021). However, no studies have reported that geraniol can ameliorate OA by regulating oxidative stress.

Nanogels are three-dimensional network structures composed of hydrophilic or amphiphilic polymer chains, which can load biologically active molecules or small molecule drugs through salt bonds, hydrogen bonds, or hydrophobic interactions (Fronza et al. 2019; Gautam et al. 2021). They have many specific advantages as a drug carrier, including easy preparation, high drug loading, stable chemical structure, swelling and shrinkage properties, easy chemical modification, and responsiveness to external environmental factors like pH, redox, and temperature (Lin et al. 2021a, b; Ribovski et al. 2021; Zhang et al. 2018). In the pathological process of OA, the joint’s pH may reach 6.0, and it contains reactive oxygen species, creating a slightly acidic and oxidative environment (Hu et al. 2021; Li et al. 2021). Based on this theory, we designed a PH/redox-responsive nanogel as a carrier for geraniol and hypothesized that it could enable geraniol to bind more fully to degenerated joints, exerting curative effects and ameliorating OA.

## Methods

### Chemicals and reagents

*N*-Isopropyl acrylamide (NIPAM), Sodium dodecyl sulfate (SDS) and potassium persulfate were purchased from Macklin (Shanghai, China). *N*-Bromoacetamide (NBA), collagenase II, dimethyl sulfoxide (DMSO), ascorbic acid and bovine serum albumin (BSA) were purchased from Sigma-Aldrich (St Louis, MO, USA). Acrylic acid (AA), l-glutamine and geraniol were purchased from Aladdin (Shanghai, China). Trypsin–EDTA, penicillin and streptomycin were purchased from Solarbio (Beijing, China). Dulbecco’s Modified Eagle’s Medium–High glucose (DMEM) and interleukin-1 beta (IL-1β) were purchased from Gibco (Gaithersburg, MD, USA). Fetal bovine serum (FBS) was purchased from Hyclone (Logan, Utah, USA). Primary antibodies against Keap1, Nrf2, HO-1, p-p65, p65, MMP13, Col2a1, ADAMTS-5, and aggrecan were purchased from Abcam (Cambridge, UK).

### Synthesis of pH/redox-responsive nanogels

The pH/redox-responsive nanogels were synthesized as follows: 1.301 g of NIPAM, 0.169 g of NBA, and 0.058 g of SDS were accurately weighed and dissolved in 200 mL of ultrapure water. The mixture was stirred with nitrogen at 70 °C for 0.5 h to remove residual oxygen. Next, 0.054 g of potassium persulfate initiator was dissolved in 2 mL of ultrapure water and added to the mixed solution. The solution was further stirred with nitrogen for 0.5 h. Then, 10 μL of AA was taken and mixed with 0.016 g of potassium persulfate and 1 mL of ultrapure water. The resulting solution was added to the mixed solution, and the reaction was continued for 5 h under nitrogen at 70 °C. After completion of the reaction, the solution was cooled to room temperature and transferred to a dialysis bag (Spectra/Por® Float-A-Lyzer® G2) with a molecular weight cut-off of 3500 Da to remove surfactants and unreacted raw materials. The mixture was dialyzed in tap water for 24 h and then transferred to ultrapure water for dialysis for 3 days, resulting in the formation of the pH/redox-responsive nanogels.

### Characterization

The chemical structure of the pH/redox-responsive nanogels was determined using Fourier transform infrared spectroscopy (FT-IR) (Perkin Elmer, USA), while X-ray diffraction (XRD) (Rigaku, Japan) was employed to analyze their crystalline structure. The size and morphology of the nanoparticles were examined using transmission electron microscopy (TEM) (Bruker, Germany).

### Drug loading

The geraniol solution (1 mg/mL) was slowly added dropwise into the nanogel solution (2.5 mg/mL), followed by sonication using an ultrasonic homogenizer (Sonics Vibra-Cell VCX 750) until the solution was completely mixed. The mixture was stirred in a dark room for 24 h to allow the geraniol to be encapsulated within the nanogels. The resulting mixture was subjected to ultrafiltration using a centrifugal filter device (Amicon® Ultra-4 Centrifugal Filter Unit) with a molecular weight cut-off of 10 kDa, and centrifuged at 5000× *g* for 10 min to remove any free geraniol and other small molecules. The purified nanogels were collected by washing the filter with ultrapure water.

### Isolation and culture of chondrocytes

This study was approved by permission from the First Affiliated Hospital of Zhejiang University School of Medicine (No. 2023.567). And the mice were supplied by Zhejiang Experimental Animal Center.

Primary chondrocytes were obtained from 3-day-old C57BL/6 mice. The articular cartilage was digested with 0.25% trypsin–EDTA for 0.5 h to remove other tissues. Then, 0.2% (w/v) collagenase II was used for digestion at 37 °C for 4 h. The chondrocytes were collected by centrifugation at 1000 rpm for 5 min and cultured in DMEM supplemented with 10% FBS, 1% penicillin/streptomycin, 1% l-glutamine, and 50 μg/mL of ascorbic acid. The cells were incubated at 37 °C in a humidified atmosphere with 5% carbon dioxide. Chondrocytes at passage 3 were used for subsequent experiments.

### Cell cytotoxicity analysis

The cytotoxicity of geraniol and nanogel was assessed using the MTT assay (Sigma-Aldrich, USA). Chondrocytes were seeded at a density of 5000 cells/well in a 96-well plate and treated with varying concentrations of geraniol and nanogel for 24 h. The MTT assay was performed by adding 20 μL of MTT solution (5 mg/mL) to each well, followed by incubation at 37 °C for 4 h. The living cells produced purple triphenylmethylamine crystals which were dissolved using DMSO, and the absorbance was measured at 490 nm using a microplate reader (Thermo Scientific Multiskan FC).

### Treatment of inflamed-chondrocytes induced by IL-1β

The chondrocytes were divided into three groups: (1) Control group, which received culture medium only; (2) IL-1β group, which was treated with 10 ng/mL IL-1β for 24 h to stimulate inflammation; (3) geraniol group, which was pre-incubated with 1 μM geraniol for 6 h and then treated with 10 ng/mL IL-1β for 24 h.

### Western blot analysis of protein expression by mouse chondrocytes

The protein expression levels of Keap1, Nrf2, HO-1, p-p65, p65, MMP13, Col2a1, ADAMTS-5, and aggrecan were measured using Western blotting. Primary chondrocytes were seeded at a density of 5 × 10^5^ cells/well in 6-well plates and lysed using ice-cold RIPA buffer containing protease and phosphatase inhibitors. After sonication and centrifugation at 12,000 rpm for 15 min at 4 °C, the supernatant was collected and its protein concentration was measured using a BCA protein assay kit (Beyotime, China). SDS-PAGE gels with varying concentrations, depending on the protein of interest, were used to load equal amounts of protein, which were then transferred onto a polyvinylidene fluoride (PVDF) membrane using a transfer apparatus. The PVDF membrane was washed with TBS-T buffer and blocked with 5% BSA at 37 °C for 1 h. Primary antibodies against Keap1, Nrf2, HO-1, p-p65, p65, MMP13, Col2a1, ADAMTS-5, and aggrecan were diluted 1:1000 in 5% BSA and added to the PVDF membrane. The membrane was incubated overnight at 4 °C with the primary antibody, then washed three times with TBS-T buffer for 10 min each time. A secondary antibody was added to the membrane, diluted in TBS-T buffer, and incubated for 1 h at room temperature. The membrane was washed three times with TBS-T buffer for 10 min each time. Protein expression levels were detected using an exposure meter after exposing the membrane to X-ray film and developing and fixing the film.

### Immunofluorescence staining

The chondrocytes were fixed with 4% paraformaldehyde for 15 min, permeabilized with 0.3% Triton X-100 for 10 min, blocked with 5% BSA for 1 h at room temperature, and then incubated with primary antibodies against p65 at 4 °C overnight. After washing three times with PBS, the cells were incubated with the secondary antibody conjugated for 1 h at room temperature. Finally, the cells were counterstained with DAPI, and images were acquired using a fluorescence microscope.

### OA-inducing anterior cruciate ligament transection (ACLT) surgery

This study was approved by permission from the First Affiliated Hospital of Zhejiang University School of Medicine (No. 2023.567). ACLT (anterior cruciate ligament transection) was performed on both knees of 8-week-old male C57BL/6 mice as previously described (Sun et al. 2022). Animals were returned to their respective cages immediately after surgery without joint fixation. Mice in the control group did not receive any treatment. Ten days after ACLT surgery, intra-articular injections of either 20 μL of geraniol in PBS (1 mg/mL) or geraniol@nanogel (equivalent to 1 mg/mL of geraniol) were administered to the mice once per week for a total of 8 weeks. Mice were housed in standard cages under controlled conditions of temperature (22 ± 2 °C) and humidity (50 ± 10%) with a 12 h light/dark cycle. Animals had free access to food and water during the experimental period. The mice were sacrificed by carbon dioxide inhalation at the end of the study, and the knee joints were harvested for further analysis. The sample size was 5 mice in each group.

### OARSI scoring of murine cartilage

The methods were conducted as previously stated in Chen et al. 2016, which includes histopathological grading using a modified version of the Chambers scoring system. This system is the standard method for grading cartilage degeneration in mice established by the OARSI Histopathology Initiative. Paraffin sections from each sample were scored after safranin-O staining. Grading was performed in four areas: medial femoral condyle, medial tibial plateau, lateral femoral condyle, and lateral tibial plateau. Three blinded observers graded each section, and the results were averaged to obtain the grades for each sample. The grades from each group of mice were then collated for analysis.

### Immunohistochemical staining

Immunohistochemical staining was performed to detect the expression of Keap1, Nrf2, HO-1, MMP13, Col2a1, ADAMTS-5, and aggrecan in cartilage tissues. Briefly, paraffin-embedded sections were deparaffinized in xylene and rehydrated in a graded series of ethanol. Antigen retrieval was performed by incubating the slides in citrate buffer (pH 6.0) at 95 °C for 15 min. The sections were then treated with 3% hydrogen peroxide for 10 min to quench endogenous peroxidase activity. After blocking with 5% bovine serum albumin, the sections were incubated with primary antibodies against Keap1, Nrf2, HO-1, MMP13, Col2a1, ADAMTS-5, and aggrecan overnight at 4 °C. The primary antibodies were detected using a horseradish peroxidase-conjugated secondary antibody and visualized using a DAB substrate kit. Finally, the sections were counterstained with hematoxylin, dehydrated, and mounted.

### Statistical analysis

Statistical analyses were performed by SPSS statistics 22.0. All data were expressed as the mean ± standard deviation (SD), and all independent experiments were repeated at least three times. One-way analysis of variance (ANOVA) was used to assess group differences. Kolmogorov–Smirnov test was used to analyze the ANOVA model residuals to check the normality for all groups. p < 0.05 was considered statistically significant.

## Results

### Geraniol inhibits chondrocyte matrix degradation induced by IL-1β

**Fig. 1:**
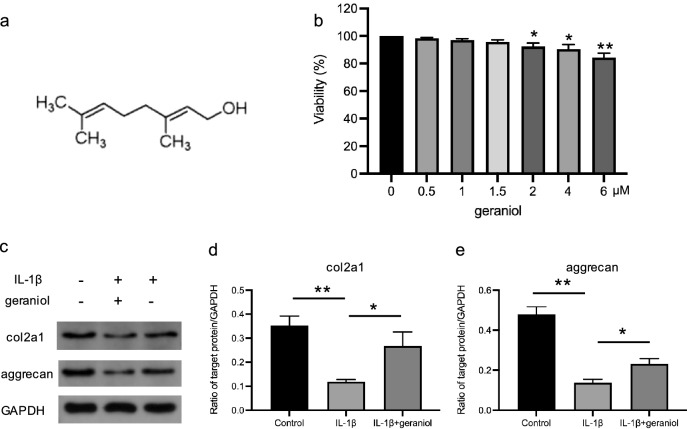
Geraniol inhibits chondrocyte matrix degradation. **a** Molecular structure of geraniol. **b** Toxicity of geraniol on chondrocytes. **c** Western blot analysis of col2a1, aggrecan protein expression levels. **d**, **e** Quantification of specific signal intensities. GADPH was used as loading control. *p < 0.05, **p < 0.01, n = 3

The molecular structure of geraniol is presented in Fig. 1a. The cytotoxicity of geraniol was first evaluated, and the results indicated that geraniol was non-toxic when the concentration was below 1.5 μM (Fig. 1b). Therefore, 1 μM was selected as the final experimental concentration. Subsequently, the effect of geraniol on chondrocyte matrix degradation was examined. The results demonstrated that IL-1β significantly induced chondrocyte matrix degradation (p < 0.01), while geraniol significantly inhibited this process (p < 0.05) (Fig. 1c–e).

### The effect of geraniol on Keap1/Nrf2/HO-1 pathway and nuclear translocation of NF-κB p65

**Fig. 2:**
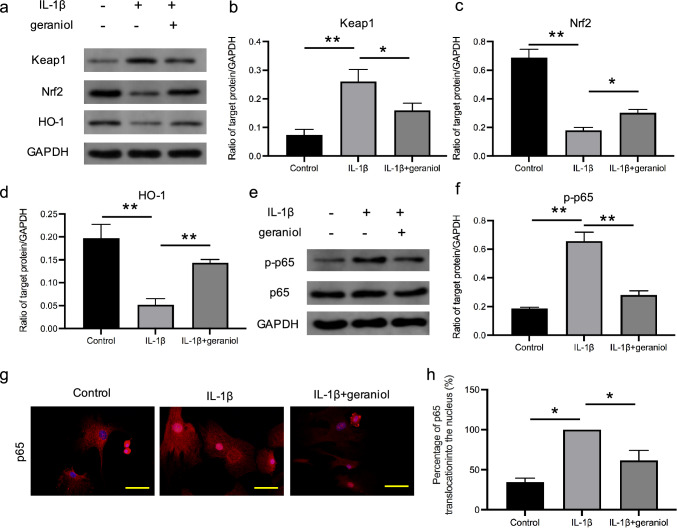
The effect of geraniol on Keap1/Nrf2/HO-1 pathway and nuclear translocation of NF-κB p65. **a** Western blot analysis of Keap1, Nrf2 and HO-1 protein expression levels. **b**–**d** Quantification of specific signal intensities. GADPH was used as loading control. **e** Western blot analysis of p65 protein expression levels. **f** Quantification of specific signal intensities. GADPH was used as loading control. **g** Fluorescent staining of NF-κB p65 translocation into the nucleus. Bar = 20 μm. **h** Quantitative analysis of the percentage of positive cells. *p < 0.05, **p < 0.01, n = 3

To explore the effect of geraniol on oxidative stress and inflammation in chondrocytes stimulated by IL-1β, we assessed the protein expressions of Keap1, Nrf2, HO-1, and p65 using Western blot and immunofluorescence. The protein expression of Keap1 was significantly lower in chondrocytes treated with geraniol compared to those treated with IL-1β alone (p < 0.05), while the protein expressions of Nrf2 and HO-1 were significantly higher in geraniol-treated chondrocytes compared to those in the IL-1β group (p < 0.05) (Fig. 2a–d). Moreover, geraniol significantly reduced the expression of p-p65 induced by IL-1β (p < 0.01) (Fig. 2e, f) and inhibited the nuclear translocation of NF-κB p65 (p < 0.05) (Fig. 2g, h). These results demonstrate that geraniol has anti-oxidative stress and anti-inflammatory effects in IL-1β-stimulated chondrocytes.

### Characterization and detection of pH/redox-responsive nanogels

**Fig. 3:**
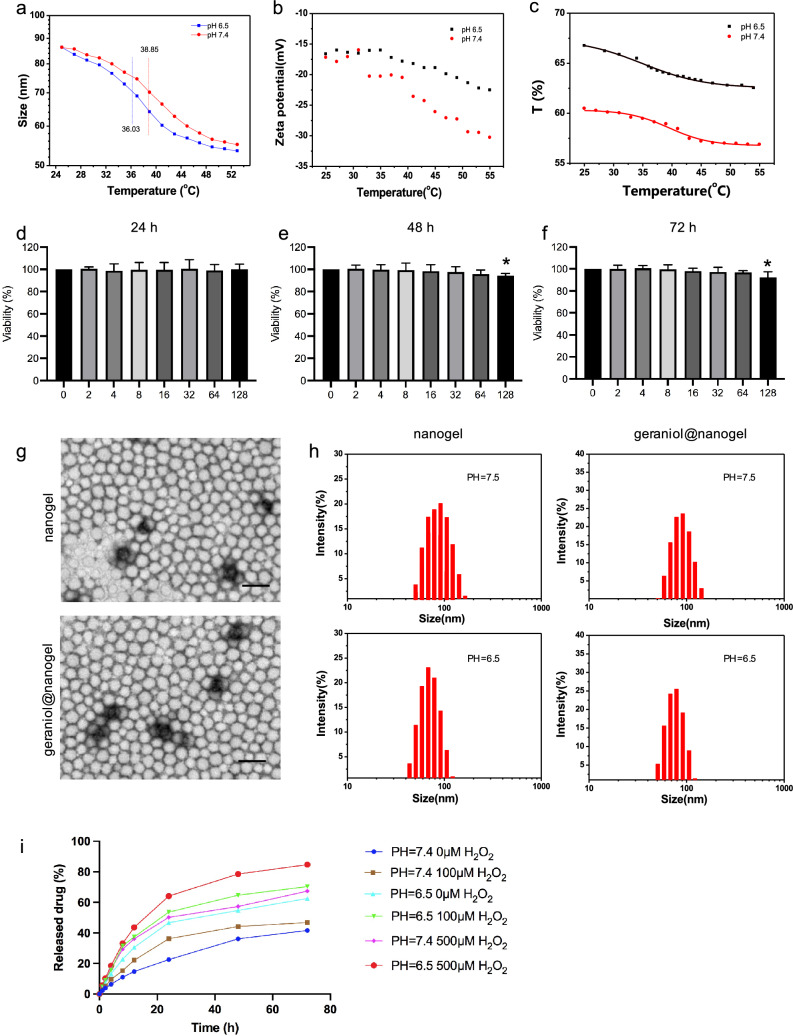
Characterization and detection of pH/redox-responsive nanogels. The particle size (**a**), Zeta potential (**b**) and transmittance (**c**) of the nanogels at pH 6.5 or 7.5 with different temperatures. Toxicity of pH/redox-responsive nanogels on chondrocytes at 24 (**d**), 48 (**e**) and 72 (**f**) h. **g** Electron microscopy was inducted to evaluate microscopic structure of nanogel and geraniol@nanogel. **h** The particle size distribution diagrams of nanogel and geraniol@nanogel at different PH. **i** Sustained release profiles of geraniol@nanogel under different conditions

We prepared pH/redox-responsive nanogels and examined the characterization of the gels. We first detected the particle size, Zeta potential and transmittance of the nanogels at pH 6.5 or 7.5 with different temperatures (Fig. 3a–c). The results showed that the particle size of the nanogels remained around 70 nm at around 37 °C. The particle size of the nanogels at pH 6.5 was smaller than that at pH 7.5, and the Zeta potential and transmittance were higher. Subsequently, we tested the toxic and side effects of the nanogels. To test the toxicity of nanogels, chondrocytes were treated with different concentrations of geraniol@nanogel (0, 2, 4, 6, 8, 16, 32, 64 and 128 μM) for 24 h. The chondrocytes were observed under a microscope, and cell viability was determined by the MTT assay. The nanogels were dissolved in PBS before use. The results showed that geraniol@nanogel had no obvious cytotoxicity to chondrocytes at concentrations up to 128 μM (Fig. 3d–f). We used nanogels to encapsulate geraniol, and observed the morphologies of blank nanogels and geraniol-loaded nanogels by electron microscopy. We found that the morphology of the nanogels before and after drug loading were regular spherical, uniform in size, and the particle size distribution was mainly concentrated around 70 nm (Fig. 3g). The results indicated that the encapsulation of geraniol had no significant effect on the morphology and particle size of the nanogel carrier. In addition, at pH 6.5 and 7.5, the particle size distribution diagrams of blank nanogels and geraniol nanogels showed that the entrapment of geraniol had no significant effect on the particle size of the nanogel carriers, but low pH will decrease the particle size of the nanogels (Fig. 3h). Finally, we tested drug release in vitro using dialysis. Briefly, we prepared the geraniol@nanogel and placed it in a dialysis bag with a molecular weight cutoff of 8,000–14,000 Da. The dialysis bag was immersed under pH6.5 and pH7.4 conditions, as well as under 0 μM, 100 μM, and 500 μM H_2_O_2_ concentrations. The release medium was collected at different time points, and the concentration of geraniol was measured by HPLC. We then plotted the cumulative release of geraniol over time to evaluate the release profile. We found that geraniol can be released effectively and controllably from nano-drug delivery systems under pH/oxidative stress stimulation (Fig. 3i).

### Geraniol@nanogel inhibits OA formation

**Fig. 4:**
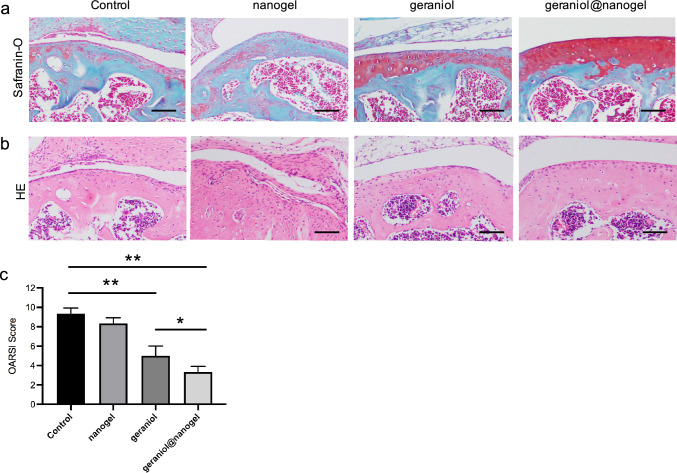
Geraniol@nanogel inhibits OA formation. Safranin O (**a**) and HE (**b**) staining of OA samples. **c** Histological score of cartilages in different groups. *p < 0.05, **p < 0.01, n = 3

In this study, we investigated the therapeutic effect of geraniol@nanogel on osteoarthritis (OA) formation in male C57BL/6 mice following ACLT surgery. The mice were divided into four groups: control, nanogel, geraniol, and geraniol@nanogel. Mice in the geraniol and geraniol@nanogel groups were treated with intra-articular injections of either 20 μL of geraniol in PBS (1 mg/mL) or geraniol@nanogel (equivalent to 1 mg/mL of geraniol) once per week for eight weeks. The nanogel group received the same volume of nanogel, while the control group received no treatment. At the end of the experiment, we sacrificed the mice and collected their knee joints for histological evaluation, which was performed using the modified OARSI scoring system, as described in “OARSI scoring of murine cartilage” section. Our results demonstrated that the geraniol@nanogel group had significantly improved histological scores compared to the control, nanogel, and geraniol groups (Fig. 4c, p < 0.05). This indicated that geraniol@nanogel had a significant repairing effect on degenerated cartilage. Additionally, we observed that the geraniol@nanogel group had slight superficial fibrillation around the cartilage surface and little decrease in the proteoglycan in the cartilage matrix, suggesting an enormous repair capacity of geraniol@nanogel for OA. Therefore, we conclude that geraniol@nanogel is a promising therapeutic agent for the treatment of OA-induced cartilage damage in mice.

### Geraniol@nanogel inhibits cartilage matrix degradation

**Fig. 5:**
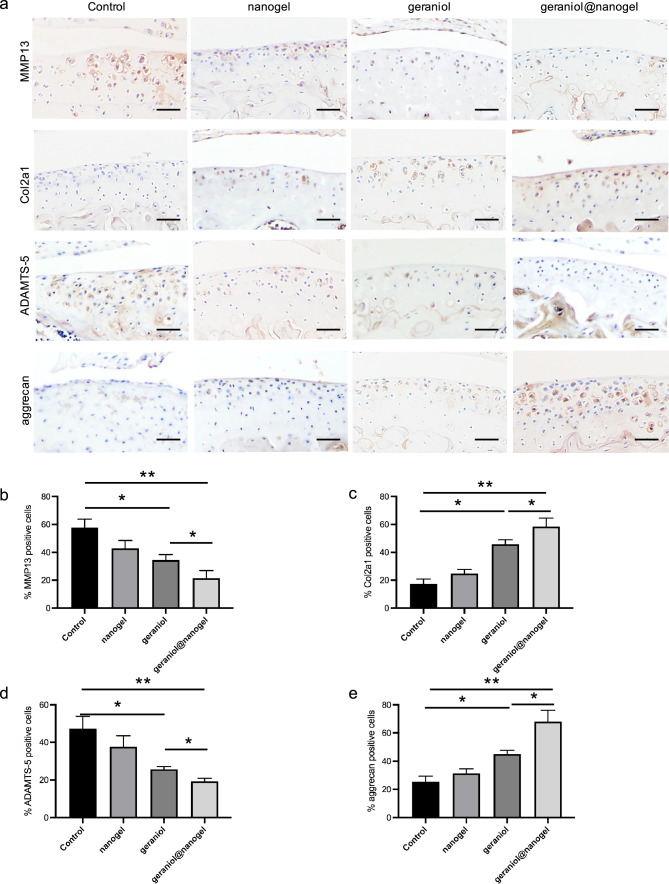
Geraniol@nanogel inhibits cartilage matrix degradation. **a** Expression of MMP13, Col2a1, ADAMTS-5 and aggrecan in different groups. Percentages of cells immune-positive for MMP13 (**b**), Col2a1 (**c**), ADAMTS-5 (**d**) and aggrecan (**e**). *p < 0.05, **p < 0.01, n = 3

We investigated the impact of geraniol@nanogel on cartilage matrix. To quantify the immunohistochemical images, we utilized ImageJ software for analysis. The immunohistochemical results indicated that the expressions of MMP13 and ADAMTS-5 in the geraniol@nanogel group were significantly reduced, while the expressions of Col2a1 and aggrecan were significantly increased, compared to the control, nanogel, and geraniol groups (p < 0.05) (Fig. 5). These findings demonstrate that geraniol@nanogel effectively inhibits cartilage matrix degradation.

### Geraniol@nanogel inhibits oxidative stress

**Fig. 6:**
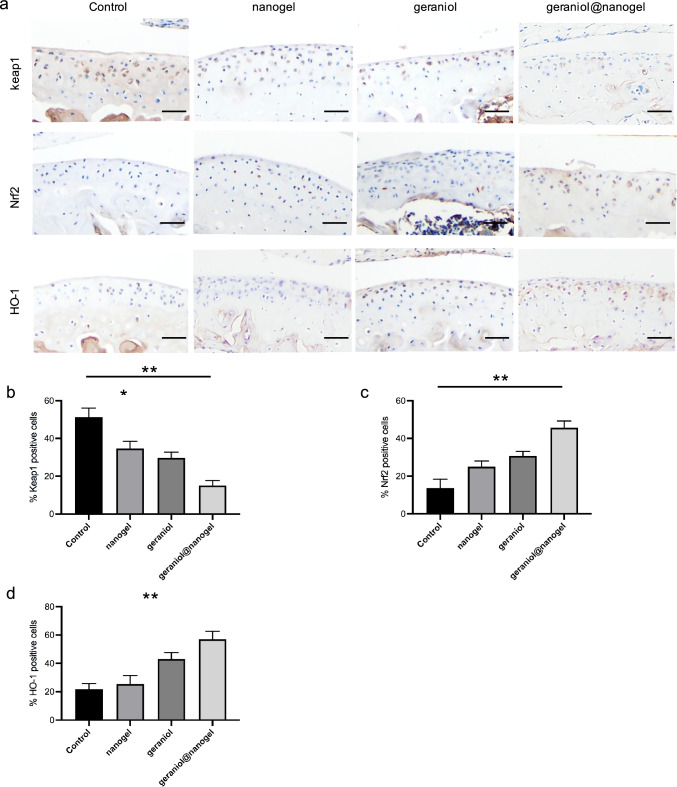
Geraniol@nanogel inhibits oxidative stress. **a** Expression of Keap-1, Nrf2, and HO-1 in different groups. Percentages of cells immune-positive for Keap-1 (**b**), Nrf2 (**c**), and HO-1 (**d**). *p < 0.05, **p < 0.01, n = 3

Furthermore, we evaluated the effect of geraniol@nanogel on oxidative stress. The results of immunohistochemistry showed that the expression of Keap1 in the geraniol@nanogel group was significantly reduced, while the expressions of Nrf2 and HO-1 were significantly increased, compared to the control, nanogel, and geraniol groups (p < 0.05) (Fig. 6). These results demonstrate that geraniol@nanogel effectively inhibits oxidative stress.

### Statistical analysis results

The results of Kolmogorov–Smirnov test and One-way ANOVA analysis were summarized in Table 1. All the data we have acquired conforms to a normal distribution. All the data, apart from the Toxicity of nanogels on chondrocytes at 24 h data, showed statistically significant results in the One-way ANOVA analysis (p < 0.05).

**Table 1:** Statistical analysis results

	Kolmogorov–Smirnov test		One-way ANOVA	
	D-value	P-value	F-value	P-value
Quantification of signal				
col2a1	0.179	0.889	24.629	1e−03
aggrecan	0.128	0.994	119.155	1.48e−05
Keap1	0.111	0.999	28.008	9.06e−04
Nrf2	0.127	0.994	152.118	7.23e−06
HO-1	0.149	0.971	42.725	2.82e−04
p-p65	0.155	0.960	112.385	1.76e−05
Percentage of positive cells	0.278	0.491	53.031	1.53e−04
Toxicity at different time				
24h	0.170	0.445	0.006	0.938
48h	0.114	0.114	7.008	0.015
72h	0.114	0.914	17.143	4.28e−04
OARSI score	0.293	0.254	47.333	1.95e−05
Percentages of cells immune-positive				
MMP13	0.193	0.693	23.72	2.46e−04
Col2a1	0.121	0.986	1.622	7.16e−06
ADAMTS-5	0.166	0.845	22.956	2.76e−04
aggrecan	0.147	0.926	44.518	2.45e−05
Keap-1	0.138	0.952	50.834	1.49e−05
Nrf2	0.145	0.931	42.513	2.92e−05
HO-1	0.153	0.903	30.548	9.89e−05

## Discussion

OA is a degenerative chronic disease of articular cartilage degenerative damage, reactive hyperplasia of articular margins, subchondral bone, synovial membrane, and other joint tissues caused by multiple factors, which has affected millions of people worldwide (Mandl 2019), yet the current diagnosis and treatment methods for OA are inadequate. Intra-articular injection is an effective modality for OA, as it allows direct delivery of the drug to the primary site of disease development (Jones et al. 2019). In recent years, various delivery systems have been reported and applied in intra-articular injection applications, such as liposomes (Chang et al. 2021), microspheres (Han et al. 2021) and hydrogels (Tao et al. 2021). However, the efficacy of drug delivery is limited, which hinders the treatment of OA.

Due to their size, most carriers are too large to effectively penetrate chondrocytes, hindering drug delivery. However, it has been demonstrated that nanoparticles (< 96 nm) can enter the dense extracellular matrix of articular cartilage (Bedingfield et al. 2021). Furthermore, rapid drug clearance from the synovial joint presents another obstacle to effective treatment. Most intra-articular drugs in OA patients are rapidly cleared within two hours of injection due to the lack of specific binding to the joint (Wang et al. 2022). Targeting specific areas of the joint can increase the residence time of the drug in the joint cavity. In addition, OA commonly exhibits an acidic pH microenvironment, which is negatively correlated with the severity of the disease, and is characterized by inflammation (Li et al. 2022a, b). To address these challenges, we developed a pH/redox-responsive nanogel as a carrier for geraniol, providing an effective delivery system for OA treatment. We hypothesized that the nanogel could penetrate the cartilage ECM and specifically bind to the joint in the acidic OA microenvironment, thus increasing the residence time of geraniol in the joint cavity and improving its efficacy. While direct binding of the nanogel to the joint was not demonstrated, our hypothesis is supported by the properties of the nanogel and the observed improvement in efficacy. Our study revealed that geraniol release from geraniol@nanogel increased with decreasing pH (pH 6.5 vs. pH 7.5), indicating that geraniol is continuously released on demand according to the severity of joint inflammation, as dictated by the pH-triggered release properties of the nanogels.

In addition to our findings, several studies have reported the therapeutic benefits of terpenoids in treating OA. For instance, artemisinin, a widely known antimalarial drug, possesses anti-inflammatory and analgesic effects in animal models of OA (Li et al. 2022a, b). Similarly, curcumin, a natural compound derived from turmeric, exhibits anti-inflammatory and antioxidant properties, making it a promising therapeutic agent for OA (Jin et al. 2022). Furthermore, nanogel-based drug delivery systems have been explored for the treatment of various diseases. For example, a recent study investigated the potential of nano-sized fluidizing liposomes loaded with paclitaxel, which allows enhanced drug permeation while maintaining localization of the drug depot, and enhances the efficacy of chemoradiotherapy (GuhaSarkar et al. 2016). Our study adds to this growing body of research by demonstrating the potential of pH/redox-responsive nanogel as an effective carrier for the delivery of geraniol to treat OA.

Moving forward, further research is needed to compare the efficacy of different drug delivery systems, including nanogel-based systems, for the treatment of OA. Additionally, future studies should explore the potential of combining different therapeutic agents or delivery systems to enhance treatment efficacy. In the long term, pH/redox-responsive nanogel-based drug delivery systems have the potential to revolutionize the treatment of OA, providing a safe and effective way to deliver therapeutic agents directly to the site of disease development. However, further evaluation of the long-term safety and efficacy of these systems is necessary before their widespread clinical use.

There are still some limitations in our study that need to be addressed in future research. For example, although our results showed the potential of geraniol@nanogel in treating OA, more studies are needed to compare its efficacy with other established treatments, such as non-steroidal anti-inflammatory drugs or hyaluronic acid injections. Moreover, although our study demonstrated the effectiveness of geraniol@nanogel in reducing oxidative stress and inflammation in OA, the long-term safety of the nanogel system requires further evaluation.

## Conclusion

In summary, our study has demonstrated that geraniol can effectively inhibit the development of OA by modulating oxidative stress and inflammation, and the pH/redox-responsive nanogel can serve as an effective carrier to enhance the anti-OA effect of geraniol. These findings suggest that the use of geraniol@nanogel as a therapeutic agent could provide a useful strategy for the treatment of OA in clinical applications.

## Data Availability

The datasets generated and/or analyzed during the current study are available from the corresponding author on reasonable request.

## References

[CR1] Ansari MY, Ahmad N, Haqqi TM (2020). Oxidative stress and inflammation in osteoarthritis pathogenesis: role of polyphenols. Biomed Pharmacother.

[CR2] Bedingfield SK, Colazo JM, Yu F, Liu DD, Jackson MA, Himmel LE (2021). Amelioration of post-traumatic osteoarthritis via nanoparticle depots delivering small interfering RNA to damaged cartilage. Nat Biomed Eng.

[CR3] Chang MC, Chiang PF, Kuo YJ, Peng CL, Chen KY, Chiang YC (2021). Hyaluronan-loaded liposomal dexamethasone-diclofenac nanoparticles for local osteoarthritis treatment. Int J Mol Sci.

[CR4] Chen P, Xia C, Mei S, Wang J, Shan Z, Lin X, Fan S (2016). Intra-articular delivery of sinomenium encapsulated by chitosan microspheres and photo-crosslinked GelMA hydrogel ameliorates osteoarthritis by effectively regulating autophagy. Biomaterials.

[CR5] Cho M, So I, Chun JN, Jeon JH (2016). The antitumor effects of geraniol: modulation of cancer hallmark pathways (review). Int J Oncol.

[CR6] Fronza BM, Rad IY, Shah PK, Barros MD, Giannini M, Stansbury JW (2019). Nanogel-based filler-matrix interphase for polymerization stress reduction. J Dent Res.

[CR7] Gautam D, Pedler MG, Nair DP, Petrash JM (2021). Nanogel-facilitated in-situ delivery of a cataract inhibitor. Biomolecules.

[CR8] GuhaSarkar S, Pathak K, Sudhalkar N, More P, Goda JS, Gota V, Banerjee R (2016). Synergistic locoregional chemoradiotherapy using a composite liposome-in-gel system as an injectable drug depot. Int J Nanomedicine.

[CR9] Guilak F, Nims RJ, Dicks A, Wu CL, Meulenbelt I (2018). Osteoarthritis as a disease of the cartilage pericellular matrix. Matrix Biol.

[CR10] Han Y, Yang J, Zhao W, Wang H, Sun Y, Chen Y (2021). Biomimetic injectable hydrogel microspheres with enhanced lubrication and controllable drug release for the treatment of osteoarthritis. Bioact Mater.

[CR11] Hu Y, Chen X, Wang S, Jing Y, Su J (2021). Subchondral bone microenvironment in osteoarthritis and pain. Bone Res.

[CR12] Huang K, Wu LD (2008). Aggrecanase and aggrecan degradation in osteoarthritis: a review. J Int Med Res.

[CR13] Jin Z, Chang B, Wei Y, Yang Y, Zhang H, Liu J (2022). Curcumin exerts chondroprotective effects against osteoarthritis by promoting AMPK/PINK1/Parkin-mediated mitophagy. Biomed Pharmacother.

[CR14] Jones IA, Togashi R, Wilson ML, Heckmann N, Vangsness CT (2019). Intra-articular treatment options for knee osteoarthritis. Nat Rev Rheumatol.

[CR15] Kang DG, Lee HJ, Kim KT, Hwang SC, Lee CJ, Park JS (2017). Effect of oleanolic acid on the activity, secretion and gene expression of matrix metalloproteinase-3 in articular chondrocytes in vitro and the production of matrix metalloproteinase-3 in vivo. Korean J Physiol Pharmacol.

[CR16] Li Z, Huang Z, Bai L (2021). Cell interplay in osteoarthritis. Front Cell Dev Biol.

[CR17] Li J, Jiang M, Yu Z, Xiong C, Pan J, Cai Z (2022). Artemisinin relieves osteoarthritis by activating mitochondrial autophagy through reducing TNFSF11 expression and inhibiting PI3K/AKT/mTOR signaling in cartilage. Cell Mol Biol Lett.

[CR18] Li M, Yin H, Yan Z, Li H, Wu J, Wang Y (2022). The immune microenvironment in cartilage injury and repair. Acta Biomater.

[CR19] Lin L, Long N, Qiu M, Liu Y, Sun F, Dai M (2021). The inhibitory efficiencies of geraniol as an anti-inflammatory, antioxidant, and antibacterial, natural agent against methicillin-resistant *Staphylococcus aureus* infection in vivo. Infect Drug Resist.

[CR20] Lin Y, Li C, Liu A, Zhen X, Gao J, Wu W (2021). Responsive hyaluronic acid-gold cluster hybrid nanogel theranostic systems. Biomater Sci.

[CR21] Ma T, Jia L, Zhao J, Lv L, Yu Y, Ruan H (2022). Ginkgolide C slows the progression of osteoarthritis by activating Nrf2/HO-1 and blocking the NF-kappaB pathway. Front Pharmacol.

[CR22] Maczka W, Winska K, Grabarczyk M (2020). One hundred faces of geraniol. Molecules.

[CR23] Mandl LA (2019). Osteoarthritis year in review 2018: clinical. Osteoarthr Cartil.

[CR24] Martel-Pelletier J, Barr AJ, Cicuttini FM, Conaghan PG, Cooper C, Goldring MB (2016). Osteoarthritis. Nat Rev Dis Primers.

[CR25] Mlost J, Kac P, Kedziora M, Starowicz K (2022). Antinociceptive and chondroprotective effects of prolonged beta-caryophyllene treatment in the animal model of osteoarthritis: focus on tolerance development. Neuropharmacology.

[CR26] Palazzo C, Nguyen C, Lefevre-Colau MM, Rannou F, Poiraudeau S (2016). Risk factors and burden of osteoarthritis. Ann Phys Rehabil Med.

[CR27] Pavan B, Dalpiaz A, Marani L, Beggiato S, Ferraro L, Canistro D (2018). Geraniol pharmacokinetics, bioavailability and its multiple effects on the liver antioxidant and xenobiotic-metabolizing enzymes. Front Pharmacol.

[CR28] Peat G, Thomas MJ (2021). Osteoarthritis year in review 2020: epidemiology & therapy. Osteoarthr Cartil.

[CR29] Pereira D, Ramos E, Branco J (2015). Osteoarthritis. Acta Med Port.

[CR30] Ribovski L, de Jong E, Mergel O, Zu G, Keskin D, van Rijn P, Zuhorn IS (2021). Low nanogel stiffness favors nanogel transcytosis across an in vitro blood-brain barrier. Nanomedicine.

[CR31] Scanzello CR, Goldring SR (2012). The role of synovitis in osteoarthritis pathogenesis. Bone.

[CR32] Sun W, Yue M, Xi G, Wang K, Sai J (2022). Knockdown of NEK7 alleviates anterior cruciate ligament transection osteoarthritis (ACLT)-induced knee osteoarthritis in mice via inhibiting NLRP3 activation. Autoimmunity.

[CR33] Tang Q, Zheng G, Feng Z, Chen Y, Lou Y, Wang C (2017). Trehalose ameliorates oxidative stress-mediated mitochondrial dysfunction and ER stress via selective autophagy stimulation and autophagic flux restoration in osteoarthritis development. Cell Death Dis.

[CR34] Tao SC, Huang JY, Gao Y, Li ZX, Wei ZY, Dawes H, Guo SC (2021). Small extracellular vesicles in combination with sleep-related circRNA3503: a targeted therapeutic agent with injectable thermosensitive hydrogel to prevent osteoarthritis. Bioact Mater.

[CR35] Thomas AC, Hubbard-Turner T, Wikstrom EA, Palmieri-Smith RM (2017). Epidemiology of posttraumatic osteoarthritis. J Athl Train.

[CR36] Wang FS, Kuo CW, Ko JY, Chen YS, Wang SY, Ke HJ (2020). Irisin mitigates oxidative stress chondrocyte dysfunction and osteoarthritis development through regulating mitochondrial integrity and autophagy. Antioxidants (basel).

[CR37] Wang Y, Ge W, Ma Z, Ji G, Wang M, Zhou G, Wang X (2022). Use of mesoporous polydopamine nanoparticles as a stable drug-release system alleviates inflammation in knee osteoarthritis. APL Bioeng.

[CR38] Younis NS, Abduldaium MS, Mohamed ME (2020). Protective effect of geraniol on oxidative, inflammatory and apoptotic alterations in isoproterenol-induced cardiotoxicity: role of the Keap1/Nrf2/HO-1 and PI3K/Akt/mTOR pathways. Antioxidants (basel).

[CR39] Younis NS, Elsewedy HS, Shehata TM, Mohamed ME (2021). Geraniol averts methotrexate-induced acute kidney injury via Keap1/Nrf2/HO-1 and MAPK/NF-kappaB pathways. Curr Issues Mol Biol.

[CR40] Zahan OM, Serban O, Gherman C, Fodor D (2020). The evaluation of oxidative stress in osteoarthritis. Med Pharm Rep.

[CR41] Zhang T, Yang R, Yang S, Guan J, Zhang D, Ma Y, Liu H (2018). Research progress of self-assembled nanogel and hybrid hydrogel systems based on pullulan derivatives. Drug Deliv.

[CR42] Zhang YF, Huang Y, Ni YH, Xu ZM (2019). Systematic elucidation of the mechanism of geraniol via network pharmacology. Drug Des Devel Ther.

[CR43] Zheng L, Zhang Z, Sheng P, Mobasheri A (2021). The role of metabolism in chondrocyte dysfunction and the progression of osteoarthritis. Ageing Res Rev.

